# Treatment experience in two adults with creatinfe transporter deficiency

**DOI:** 10.1016/j.ymgmr.2021.100731

**Published:** 2021-02-22

**Authors:** Jack Schjelderup, Sigrun Hope, Christian Vatshelle, Clara D.M. van Karnebeek

**Affiliations:** a Department of Habilitation for adults, Haukeland University Hospital, Bergen, Norway; b Department of Neurohabilitation, Oslo University Hospital, Oslo, Norway; c NORMENT, KG Jebsen Centre for Psychosis Research, University of Oslo, Oslo, Norway; d Strusshamn Legesenter, Askøy, Norway; e Department of Pediatrics, Amsterdam University Medical Centres, Amsterdam, the Netherlands; f Dept of Pediatrics - Metabolic diseases, Radboud University Medical Center, Nijmegen, the Netherlands

**Keywords:** Intellectual disability, Adult, Creatine transporter deficiency, Betaine, Taurine, Glycine, Arginine, Creatine, Supplementation, Treatment

## Abstract

**Background:**

Creatine transporter deficiency (CTD) is an X-linked form of intellectual disability (ID) caused by *SCL6A8* mutations. Limited information exists on the adult course of CTD, and there are no treatment studies in adults.

**Methods:**

We report two half-brothers with CTD, 36 and 31 years at intervention start. Their clinical phenotypes were consistent with CTD, and intervention was indicated because of progressive disease course, with increased difficulties speaking, walking and eating, resulting in fatigue, and malnutrition. We therefore performed treatment trials with arginine, glycine and a proprietary product containing creatine and betaine, and then a trial supplementing with betaine alone.

**Results:**

In the older patient, glycine and arginine were accompanied by adverse effects, while betaine containing proprietary product gave improved balance, speech and feeding. When supplementation stopped, his condition deteriorated, and improved again after starting betaine supplement. Betaine supplementation was also beneficial in the younger patient, reducing his exhaustion, feeding difficulties and weight loss, making him able to resume his protected work.

**Discussion & conclusion:**

We report for the first time that betaine supplement was well tolerated and efficient in adults with CTD, while arginine and/or glycine were accompanied by side effects. Thus, betaine is potentially a new useful treatment for CTD patients. We discuss possible underlying treatment mechanisms. Betaine has been reported to have antagonistic effect on NKCC1 channels, a mechanism shared with bumetanide, a medication with promising results in both in autism and epilepsy. Further studies of betaine's effects in well-designed studies are warranted.

## 1 Introduction

*Creatine Transporter Deficiency (CTD)* is an X-linked disorder first described by G. S. Salomons et al. in 2001 [1]. Its prevalence was estimated to 0.25–1% in males with intellectual disabilities (ID) [[2], [3], [4]], while 0,024% of females were heterozygous carriers [5]. Most adult patients have moderate to severe ID. [6] . Speech and language development are especially delayed. Behavior problems (85%), epilepsy (59%), motor dysfunction (58%) and gastrointestinal problems (35%) are common symptoms [6].

We found descriptions of 10 adult CTD patients: Two males of 27 and 40 years had a stationary, non-progressive condition [7]. Three brothers died early, one at 21 years from tuberculosis, and two around 40 and 60 years from unknown cause [6]. Of two patients at age 70, one had severe ID, a myopathic face with ptosis, open mouth and external ophthalmoplegia and one had ID, Parkinsonism, upward gaze paresis, expressionless face, hanging mouth and hanging shoulders [8]. Three patients aged 53–66 had low stature, midface hypoplasia, hyperextensible joints, hypotonia, gastrointestinal problems, seizures, speech problems, behavior disturbance, intellectual disability and movement disorder [9].

*CTD* is caused by mutations in the SLC6A8 gene of the X chromosome, giving a dysfunctional sodium-chloride creatine transporter [10]. Because of the blood-brain barrier's limited creatine permeability, the creatine transporter is critical for creatine supply to brain cells. Two enzymes are involved in endogenous creatine synthesis. Arginine:glycine amidinotransferase (AGAT) converts glycine and arginine to guanidinoacetate (GAA) and ornithine. Guanidinoacetate *N*-methyltransferase (GAMT) transfers a methyl group from s-adenosylmethionine (SAM-e) to GAA, forming creatine. However, AGAT and GAMT are rarely co-expressed within the same brain cell. GAA uses the *SLC6A8* creatine transporter for transport into GAMT-expressing cells [11,12]. Therefore, CTD will also affect endogenous creatine synthesis in the brain.

Dunbar et al. [13] recommended oral supplementation with creatine (400 mg/kg/day), arginine (400 mg/kg/day), and glycine (150 mg/kg/day) for CTD patients. This treatment gave a moderate favorable response in 1/3 of CTD patients treated, but all males benefiting from this treatment were aged under 9 years. One child showed further improvement when SAM-e was added to the three mentioned supplements [14].

The effect of supplementation initiated in adulthood remains unknown. Therefore, we chose to describe our trials of supplementation given to two adult patients who are briefly described in Table 1.

## 2 Case presentation – patient no. 1

### 2.1 Background information

His mental and motor development were delayed from infancy. ID was diagnosed at age 4 years. At age 9, epilepsy was diagnosed. He had generalized tonic-clonic (GTC) seizures and an epileptiform EEG pattern. He was restless and had behavioral problems. From young adult age, he lived in a community home for persons with ID. He had sheltered outdoor work, working with much energy and diligence. He participated in sports activities like soccer. His memory and language were good. He showed anger and violence if caregivers limited his activities.

In his thirties, GTC seizures increased. He was anxious and depressed, with self-injury episodes. On MRI scan, his brain appeared normal, but his tongue impressed as atrophic.

## 3 Diagnostic confirmation

Because his maternal half-brother (patient no. 2) and their cousin (son of mother's sister) also had ID, one suspected an X-linked disorder. On linkage/segregation analysis, all three men shared a dinucleotide deletion (c.1006_1008del, p.Asn336del) in Xq28, comprising the *SLC6A8* gene, confirming his CTD diagnosis at age 32. His mutation was inherited from his mother, who was heterozygote for the condition, but apparently did not have intellectual disability.

## 4 Progressive disease course

At age 34, eating became slower. He was severely fatigued, needing to sleep much. He stopped talking and became unable to work. From age 32 to 35, body weight declined from 61.5 to 54 kg, giving a Body Mass Index (BMI) of 16.1 (normal range 18.5–25). At age 36, artificial nutrition or feeding through a PEG device were considered, but found unfeasible. Life expectancy without intervention seemed poor.

### 4.1 Baseline before treatment at age 36 years 5 months

#### 4.1.1 Clinical/neurological examination

He was apparently underweight. No dysmorphic features. No eye contact and no response to verbal instructions. Speech incomprehensible. Intermittent divergent strabismus on the right eye, normal ocular movements. Muscles slender; strength and tone unremarkable in all extremities. Twisting movements of upper extremities. He was rocking back and forth when sitting. Deep tendon reflexes and plantars normal. Gait unsteady, sometimes hitting walls.

**Medication** included Sodium valproate enterotablets 300 mg + 300 mg + 600 mg daily.

#### 4.1.2 Biochemical testing

S-Creatinine: 28 μmol/l (reference 60–105) S-CRP: 29 mg/ml (ref. < 5). S-Valproate: 553 μmol/l (300–700). Other hematologic and biochemical parameters and thyroid function normal.

**MR spectroscopy** before supplementation start would have had to be performed under general anesthesia. We concluded that the complication risk was too high.

Cognitive testing was unfeasible due to lack of cooperation.

## 5 Supplementation

### 5.1 First treatment trial: Oral arginine, glycine and betaine-containing proprietary blend

We gave 22 g of arginine and 8 g of glycine daily according to recommendations [13], but we replaced L-creatine with 14 g daily of a proprietary blend which according to its manufacturer contained cyclocreatine phosphate, betaine anhydrous, creatine phosphate, taurine and glycocyamine (=guanidinoacetate). (This information later appeared to be misleading.) The manufacturer was unwilling to disclose the concentration of the individual ingredients because this was a proprietary product.

After 6 weeks, his balance was poor, causing repeated falls and hematomas. Feeding was extremely difficult. We evaluated this treatment as non-effective, probably causing adverse effects. GAA, which was a constituent in the proprietary blend, is known to be toxic for brain cells. Glycine has been associated with nausea and vomiting [15,16], and arginine may cause hypotension [17]. Our hypothesis was that his symptoms were caused by arginine and/or glycine. Both were discontinued.

### 5.2 Second treatment trial: Proprietary blend alone

After 2 weeks, food intake and balance had improved and falls decreased.

After 9 1/2 weeks, he interacted more with caregivers, opening his mouth when being fed. He started speaking again, usually a couple of succeeding, appropriate words. He went outdoors for accompanied walks. When visited, he gave good eye contact and a handshake. Gait was stiff, but rapid with good balance. He was able to avoid obstacles. Because his condition was improved after the discontinuation of arginine and glycine, while treatment with proprietary blend continued, we concluded that arginine and/or glycine had been giving adverse effects.

However, after 5 months of propriety blend, his balance had deteriorated, and eating difficulties relapsed, giving weight loss from 55 to 52–53 kg. Proprietary blend dose was increased to 21 g.

5 weeks later, his appetite had improved, body weight was 55 kg, and speech a little better than baseline. He participated in leisure activities like dancing and going to a club. A considerable day-to-day function variation was present, but his average functioning was clearly improved from baseline. GTC frequency was unchanged – just above two seizures/month.

### 5.3 Relapse of symptoms after termination of supplementation

After 10 months of monotherapy, the proprietary blend became unavailable, and supplementation stopped.

4 months after the discontinuation, he was more fatigued, and eating was more difficult. His balance was deteriorated. GTC frequency was 4/month.

One year after discontinuation, his physical endurance capacity was poor, and he became unsteady and had to sit or lie down to rest after walking for 10–15 min. He spent much time resting. Food and drink intake were variable. His body weight had fallen from 58 kg shortly after termination to 55 kg.

### 5.4 Content of proprietary blend

10 months after discontinuation of the proprietary blend, we received information about an analysis of the contents of the proprietary blend, which was later published [18]. The constituents were trimethylglycine (betaine) 46%, creatinine 36%, creatine 11%, GAA 4%, taurine 4% and no cyclocreatine.

**Fig. 1:**
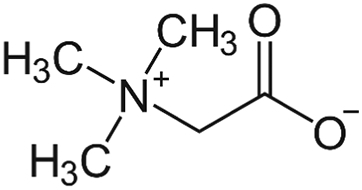
Betaine (from en.wikipedia.org).

**Betaine** (Fig. 1) is a methyl group donor, commonly used for treating homocystinuria. Betaine donates a methyl group to homocysteine, forming dimethylglycine and methionine. This increase in methionine levels has caused brain edema in some patients [19].

**Table 1:** Descriptions at start of treatment.

	Patient 1	Patient 2
Age at start of betaine	36	31
Diagnosis	F79.1 Intellectual Disability with behavioral problems	F71.0 Moderate intellectual disability F84.0 Autistic Disorder

We believed that betaine was the most important constituent for the proprietary blend's effect. Creatinine is a waste product and therefore an unlikely candidate. Creatine was unlikely because of the patient's creatine transporter defect and low dosage (2.3 g/day vs. recommended 21.6 g). GAA was unlikely because of deterioration when taking its precursors glycine and arginine, although we could not exclude its possible role. Taurine has been associated with beneficial effect on peripheral muscle function was therefore also a possible candidate for giving a beneficial effect, but we considered it unlikely because of its low concentration in the blend.

### 5.5 Third treatment trial: Betaine supplementation

This started at age 37 ½ years, about 1 year after discontinuation of the proprietary blend. The dosage increased gradually from 2.5 to 10 g daily, corresponding to the betaine fraction of 21 g of the proprietary blend.

7 weeks after treatment start, at dosage 7 g, he could eat quite well in some meals. Body weight increased to 56 kg.

After 1 year and 7 months with betaine, he ate well at more than half of the meals. Body weight was 58 kg. When walking, a caregiver held his hand. He seldom talked, but could give adequate comments. He rested 2–4 h daily, and he took part in leisure activities like going to a club for people with ID. He gave adequate eye contact. Monthly GTC frequency was 5.2. As seen in Table 2, the levels of creatine kinase (CK) appeared to be a little lower in periods of betaine treatment. The cause of this change is unclear, but one could hypothesize that betaine may have had a protective effect on muscle cells.

**Table 2:** Summary of biochemical tests. "*L" indicates values below reference range.

Selected biochemical biomarkers	Patient 1				Patient 2	
	Before treatment	With betaine-containing proprietary blend	Interlude without treatment	With betaine	Before betaine	With betaine
Creatinine (reference values 60–105 μmol/l)	28 μmol/l*L	30–36 μmol/l*L	26–28-30 μmol/l *L	26–31-31 μmol/l*L	41 μmol/l*L	36–29 μmol/l*L
CK (ref. 50–400 U/L)	83	29–39-39 U/l*L	38–46 U/l*L	24–33 U/l*L		
Methionine μmol/l. (ref. 18–33 μmol/l)				2 months: 35.8 μmol/l. 7 months: 39 μmol/l.	28.9 μmol/l.	After 6 months 32,8 μmol/l
Homocysteine (ref. <15 μmol/l)				6,7 μmol/l	13,0 μmol/l	
Methylmalonic acid (ref. <0,26 μmol/l)				0,11 μmol/l	0,19 μmol/l	
Analyses for methionine, homocysteine and methylmalonic acid were performed by Bevital, Bergen, Norway. Other analyses were performed at Haukeland University Hospital, Bergen, Norway.						

## 6 Case presentation – patient no. 2

He was the maternal half-brother of patient no. 1, being 7 years younger. He had moderate ID. At age 12, he had a GTC epileptic seizure. He started treatment with sodium valproate and became seizure-free. Medication was discontinued some years later without relapse of epilepsy. At age 25, CTD was diagnosed.

In young adulthood, he moved to a community home for people with ID. He started in a sheltered work, using a machine to gather thin planks in bundles.

In his late twenties, eating had become very slow, and he lost 2 kg weight during a couple of years. He often became exhausted and needed more help from his caregivers. He had increased obsessive behavior and self-mutilation. At age 31, he was referred to an outpatient clinic for neurohabilitation.

### 6.1 Clinical/neurological examination

Body weight 50 kg and height 183 cm gave a low BMI of 15.8. He kept his eyes almost closed. He had several small self-inflicted wounds. Speech was a little blurred, but he gave adequate answers to questions regarding daily life. Muscles were slender; power in extremities was somewhat weak. Otherwise, general and neurologic examinations were normal. On observation while eating, he used a teaspoon, eating very slowly.

### 6.2 Psychologic testing

A psychologist tested the patient and interviewed family and caregivers. ICD-10 diagnoses F71.0 Moderate intellectual disability and F84.0 Autistic disorder were confirmed.

At age 31 ½, his feeding difficulties increased. Meals lasted for up to 2 h. Body weight decreased 2.5 kg during 3 weeks to 46 kg (BMI 14.5). He needed liquid calorie rich dietary supplementation. He got a sick leave from his protected work in order to get enough time for eating. Caregivers sat down with him during meals to help him focus on eating. At this time, his half-brother had taken betaine for 3 months and seemed to have a beneficial effect. Therefore, we suggested a trial with betaine supplementation.

### 6.3 Treatment trial with betaine

He received 4 g daily, increasing gradually to 8 g after 2 months. Feeding improved rapidly, and after 5 weeks, weight had increased 2–3 kg. After 4 months, weight was stable at 50 kg. He had resumed his protected work, 80% of full time, and performed well in his job. His caregivers often had to remind him to eat and drink. He appeared to thrive better. Self-mutilation had decreased. Speech was clear. At 15 months of betaine supplement, his condition was stable; his weight was 52.5 kg (BMI 16.6). He worked 80% and had many leisure activities.

## 7 Discussion

In this case presentation, we have reported for the first time a beneficial effect from betaine supplementation in adults with CTD. Both brothers had a progressive disease course from age around 30 years. The most serious problems for both was general fatigue, reduced motor speed and feeding/swallowing difficulties. PEG treatment would probably have been necessary without betaine, but would have required strong coercive measures. We believe that the deterioration experienced by both patients was not solely caused by CTD in itself, but at a certain point in the disease course, both patients had increasing problems related to food intake, causing malnutrition, which in turn has worsened the clinical picture.

Betaine was well tolerated, exerting a positive effect on verbal and motoric functions including feeding and balance. On betaine treatment, both have a stable situation with relatively good QOL. Both patients had a moderate increase in methionine levels, but no suspicion of brain edema. For patient 2, improved meal routines (see below) may also have contributed to his improvement.

In patient 1, his symptoms improved with betaine–containing supplement alone, and progressed when the supplement stopped. When betaine supplement started one year later, his condition improved again. Tongue atrophy (seen in MRI) may also have aggravated his eating problems. His clinical status and ADL functions are considerably improved compared to the situation at intervention start.

For patient 2, his weight loss has been reversed, his self-injurious behavior has decreased, and he has resumed his work. Beside betaine, an important factor for his weight gain was that caregivers facilitated the eating situation and focused on the importance of regular meals.

The first case is also important because it is the first CTD patient where a possible deteriorating effect of arginine and/or glycine has been described.

The mechanism of betaine's assumed favorable effect is unknown. We do not know whether betaine influences the cell creatine content in itself or its effects are more aspesific. However, we would like to present some hypotheses.

First, betaine may have effect in CTD by modulating GABA-transmission[20]. Betaine has been reported to have an antagonistic effect on NKCC1 channels [21], which also influences GABAergic neurotransmission [22]. Inhibiting NKCC1 is a mechanism shared with bumetanide [23], a well-known diuretic medication that in recent years has been found to influence GABAergic transmission, and thereby it has been found promising in treatment of several brain conditions, including autism [24], and epilepsy [23]. NKCC1 inhibition by bumetanide has also been tried with success in other rare neurodevelopmental disorders fragile X syndrome [25] and tuberous sclerosis [26] .

Second, betaine's properties as an osmolyte may be of importance, as betaine has similarities with creatine in being an osmolyte [27,28]. Osmotic properties are thought to be one of the central mechanism behind bumetanide's efficacy in treating brain disorders [29] [30,31]. Thus, it could be speculated that the lack of intracellular creatine in CTD may result in inefficient osmolyte regulation, and that betaine supplementation replaces the lacking creatine and thereby improves the neuronal adaption to salinity changes, edema or cellular dehydration. Betaine has osmolyte properties that even makes it act as a “chemical chaperone” increasing the stability of cell and membrane proteins [32,33].

Fourth, it is possible that betaine has some effect through modifying methylation [28,34]**.**

Methylation of GAA by GAMT to form creatine is a rate-limiting step in the creatine synthesis by neurons. Betaine could stimulate this by donating methyl groups to SAMe, which donates a methyl group to GAA to form creatine. This might reduce the burden when body demands more methyl groups for creatine synthesis. Similar mechanisms may be responsible for a beneficial effect of both betaine and s-adenosyl methionine (SAMe). However, as creatine and GAA share the same transporter [11], one would not expect GAA to enter the GAMT-expressing cells in patients suffering from CTD. Still, it cannot be excluded that there is some rest function in the creatine transporter, and that increased endogenous synthesis improves the condition slightly. Furthermore, it is possible that CTD increases the need for methylation agents in general, as creatine supplementation has been found to reduce the need for other methylation agents [34]. Thus, it is likely that betaine may have a positive effect in CTD by improving methylation capacity for other reactions than those directly involved in creatine production. Betaine's effect on muscle may be also of importance, as animal studies have shown that muscles growth improves with betaine [35], which potentially could have had a positive impact on our patients fatigue and weight loss.

To summarize, betaine has several properties that make it likely that it will have a beneficial effect in CTD, especially the properties as an osmolyte, a down regulator of the NKCC1 channel and an influencer of GABAergic transmission. These properties are similar to the properties of bumetanide, a promising new medication for treatment of autism and epilepsy, which are common symptoms of CTD. Further research is needed, however, to elucidate the role of betaine in CTD.

### 7.1 Strengths

During all treatment periods after discontinuation of arginine and glycine, the patients' conditions improved, and the first patient's condition worsened after stopping the supplement.

### 7.2 Limitations

We acknowledge a number of limitations of our study. The first and main limitation was that the study was open, without a placebo control. Second, the supplementation was initiated and carried out without MR spectroscopy. Third, formal cognitive testing was only performed once (in patient 2). Thus, we can only use subjective parameters to evaluate the effect. Fourth, we do not know whether any of the other constituents of the proprietary blend had an additional beneficial effect that was lost when using betaine alone. Fifth, for patient 2, his caregivers' improving of meal routines may be an additional or alternate mechanism for the patient's improvement.

## 8 Conclusion

Further studies on the possible mechanism of betaine as well as evaluation of its disease course-modifying effects in a larger number of patients following a well-designed study protocol are warranted.

## Funding

CvK's work is supported by Stichting Metakids.

## References

[1] Salomons G.S. (2001). X-linked creatine-transporter gene (SLC6A8) defect: a new creatine-deficiency syndrome. Am. J. Hum. Genet..

[2] Arias A. (2007). Creatine transporter deficiency: prevalence among patients with mental retardation and pitfalls in metabolite screening. Clin. Biochem..

[3] Clark A.J. (2006). X-linked creatine transporter (SLC6A8) mutations in about 1% of males with mental retardation of unknown etiology. Hum. Genet..

[4] Rosenberg E.H. (2004). High prevalence of SLC6A8 deficiency in X-linked mental retardation. Am. J. Hum. Genet..

[5] DesRoches C.L. (2015). Estimated carrier frequency of creatine transporter deficiency in females in the general population using functional characterization of novel missense variants in the SLC6A8 gene. Gene.

[6] van de Kamp J.M. (2013). Phenotype and genotype in 101 males with X-linked creatine transporter deficiency. J. Med. Genet..

[7] Sempere A. (2009). Creatine transporter deficiency in two adult patients with static encephalopathy. J. Inherit. Metab. Dis..

[8] Kleefstra T. (2005). Progressive intestinal, neurological and psychiatric problems in two adult males with cerebral creatine deficiency caused by an SLC6A8 mutation. Clin. Genet..

[9] Hahn K.A. (2002). X-linked mental retardation with seizures and carrier manifestations is caused by a mutation in the Creatine-transporter gene.Pdf>. Am. J. Hum. Genet..

[10] Braissant O. (2011). Creatine deficiency syndromes and the importance of creatine synthesis in the brain. Amino Acids.

[11] Beard E., Braissant O. (2010). Synthesis and transport of creatine in the CNS: importance for cerebral functions. J. Neurochem..

[12] Hanna-El-Daher L., Braissant O. (2016). Creatine synthesis and exchanges between brain cells: what can be learned from human creatine deficiencies and various experimental models?. Amino Acids.

[13] Dunbar M. (2014). Treatment of X-linked creatine transporter (SLC6A8) deficiency: systematic review of the literature and three new cases. Mol. Genet. Metab..

[14] Jaggumantri S. (2015). Treatment of Creatine Transporter (SLC6A8) Deficiency With Oral S-Adenosyl Methionine as Adjunct to L-arginine, Glycine, and Creatine Supplements. Pediatr. Neurol..

[15] Hahn R.G., Shemais H., Essen P. (1997). Glycine 1.0% versus glycine 1.5% as irrigating fluid during transurethral resection of the prostate. Br. J. Urol..

[16] Strzelecki D., Rabe-Jablonska J. (2011). Changes in positive and negative symptoms, general psychopathology in schizophrenic patients during augmentation of antipsychotics with glycine: a preliminary 10-week open-label study. Psychiatr. Pol..

[17] Jun T., Wennmalm A. (1994). L-arginine-induced hypotension in the rat: evidence that NO synthesis is not involved. Acta Physiol. Scand..

[18] Tao D. (2019). Facile high-performance liquid chromatography mass spectrometry method for analysis of Cyclocreatine and Phosphocyclocreatine in complex mixtures of amino acids. J. Agric. Food Chem..

[19] Yaghmai R. (2002). Progressive cerebral edema associated with high methionine levels and betaine therapy in a patient with cystathionine beta-synthase (CBS) deficiency. Am. J. Med. Genet..

[20] Knight L.S. (2017). Betaine in the brain: characterization of Betaine uptake, its influence on other Osmolytes and its potential role in Neuroprotection from osmotic stress. Neurochem. Res..

[21] Schliess F. (2002). Expression and regulation of the Na(+)/K(+)/2Cl(−) cotransporter NKCC1 in rat liver and human HuH-7 hepatoma cells. Arch. Biochem. Biophys..

[22] Wang D.D., Kriegstein A.R. (2011). Blocking early GABA depolarization with bumetanide results in permanent alterations in cortical circuits and sensorimotor gating deficits. Cereb. Cortex.

[23] Lykke K. (2016). The search for NKCC1-selective drugs for the treatment of epilepsy: structure-function relationship of bumetanide and various bumetanide derivatives in inhibiting the human cation-chloride cotransporter NKCC1A. Epilepsy Behav..

[24] James B.J., Gales M.A., Gales B.J. (2019). Bumetanide for autism Spectrum disorder in children: a review of randomized controlled trials. Ann. Pharmacother..

[25] Lemonnier E. (2013). Treating fragile X syndrome with the diuretic bumetanide: a case report. Acta Paediatr..

[26] van Andel D.M. (2020). Effects of bumetanide on neurodevelopmental impairments in patients with tuberous sclerosis complex: an open-label pilot study. Mol. Autism..

[27] Alfieri R.R. (2006). Creatine as a compatible osmolyte in muscle cells exposed to hypertonic stress. J. Physiol..

[28] Figueroa-Soto C.G., Valenzuela-Soto E.M. (2018). Glycine betaine rather than acting only as an osmolyte also plays a role as regulator in cellular metabolism. Biochimie.

[29] Baran H. (1987). Effect of mannitol treatment on brain neurotransmitter markers in kainic acid-induced epilepsy. Neuroscience.

[30] Kharod S.C., Kang S.K., Kadam S.D. (2019). Off-label use of Bumetanide for brain disorders: an overview. Front. Neurosci..

[31] Walcott B.P., Kahle K.T., Simard J.M. (2012). Novel treatment targets for cerebral edema. Neurotherapeutics.

[32] Diamant S. (2001). Chemical chaperones regulate molecular chaperones in vitro and in cells under combined salt and heat stresses. J. Biol. Chem..

[33] Harries D., Rosgen J. (2008). A practical guide on how osmolytes modulate macromolecular properties. Methods Cell Biol..

[34] Taes Y.E. (2007). Lowering methylation demand by creatine supplementation paradoxically decreases DNA methylation. Mol. Genet. Metab..

[35] Chen R. (2019). Effects of dietary supplementation with betaine on muscle growth, muscle amino acid contents and meat quality in Cherry Valley ducks. J. Anim. Physiol. Anim. Nutr. (Berl.).

